# *Rattus norvegicus* BN/SHR liver and heart left ventricle ribosomal RNA depleted directional RNA sequencing

**DOI:** 10.1186/s13104-017-2716-4

**Published:** 2017-08-11

**Authors:** Emanuel Wyler, Sebastiaan van Heesch, Eleonora Adami, Norbert Hubner, Markus Landthaler

**Affiliations:** 1 Berlin Institute for Medical Systems Biology, Max Delbrück Center for Molecular Medicine, 13125 Berlin, Germany; 2 Cardiovascular and Metabolic Sciences, Max Delbrück Center for Molecular Medicine, 13125 Berlin, Germany

**Keywords:** Rat, RNA, Sequencing, BN-*Lx*, SHR, Liver, Heart

## Abstract

**Objective:**

The spontaneously hypertensive rat strain is a frequently used disease model. In a previous study, we measured translational efficiency from this strain and BN-*Lx* animals. Here, we describe long RNA sequencing reads from ribosomal RNA depleted samples from the same animals. This data can be used to investigate splicing-related events.

**Results:**

RNA was extracted from rat liver and heart left ventricle from BN-*Lx* and SHR/Ola rats in biological replicates. Ribosomal RNA was removed and the samples subjected to directional high-throughput RNA-sequencing. Read and alignment statistics indicate high quality of the data. The raw sequencing reads are freely available on the NCBI short read archive and can be used for further research on tissue and strain differences, or analysed together with other published high-throughput data from the same animals.

## Introduction

The inbred rat strain SHR/Ola (spontaneously hypertensive rat) is frequently used to study cardiovascular and metabolic diseases [1]. In a previous study, we investigated translational regulation in this strain compared to the BN-*Lx* reference strain [2], in which small mRNA fragments were sequenced alongside the ribosome-protected fragments. However, since these sequencing reads are on average only 40 nucleotides long, we proceeded to generate also long RNA sequencing reads from the same animals. This enables investigation of features that are more depending on mapping length, such as splicing, circular RNA detection, or variant calling.

## Main text

Total RNA was isolated from frozen, pulverized liver and heart left ventricle. Here, we describe the ribosomal RNA-depleted RNA sequencing obtained from these samples. An overview with SRA and BioSample identifiers is given in Table 1. The animal identifiers here (BN-03 etc.) correspond to the previously used identifiers [2], which can be found in the BioProject https://www.ncbi.nlm.nih.gov/bioproject/PRJEB7498.

**Table 1:** Overview of sequenced samples

Description	BioSample ID	SRA ID
BN-03 liver	SAMN04543787	SRS1884865
BN-03 heart left ventricle	SAMN04543786	SRS1884868
BN-04 liver	SAMN04543789	SRS1884867
BN-04 heart left ventricle	SAMN04543788	SRS1884866
SHR-02 liver	SAMN04543791	SRS1884869
SHR-02 heart left ventricle	SAMN04543790	SRS1884870
SHR-03 liver	SAMN04543793	SRS1884871
SHR-03 heart left ventricle	SAMN04543792	SRS1884872

**Fig. 1:**
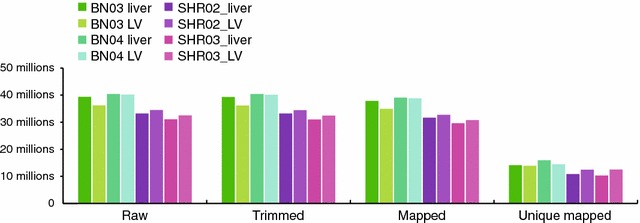
Number of reads of the eight RNA sequencing samples described here. Shown are, from *left* to *right*: raw read numbers; reads after adapter trimming and removal of reads shorter than 18 nucleotides; reads mapping to the rat genome/transcriptome; unique reads mapping to the rat genome/transcriptome

The Illumina Ribo-Zero kit (Illumina Cat.-No. MRZH11124) was applied to remove ribosomal RNA and the sequencing libraries were prepared using the Illumina TruSeq Stranded mRNA Library Prep Kit (Illumina Cat.-No. RS-122-2101). The raw sequencing reads can be downloaded from the NCBI SRA (short read archive) under the accession number SRP095829 (https://www.ncbi.nlm.nih.gov/sra/?term=SRP095829), or accessed through the NCBI BioProject PRJNA314751 (https://www.ncbi.nlm.nih.gov/bioproject/PRJNA314751). Per sample, 30–40 million reads were obtained. The adapter sequence GATCGGAAGAGCACACGT was trimmed using Flexbar [3] and trimmed reads with 18 nucleotides or longer were aligned to version rn6 of the rat genome in conjunction with the Ensembl annotation version 81 using tophat2 [4]. The numbers of raw, trimmed, aligned, and unique aligned reads are shown in Fig. 1. The absolute and relative numbers of aligned and unique aligned reads are shown alongside the number of reads mapping to the rat 45S ribosomal RNA in Table 2.

**Table 2:** Alignment statistics

Description	Aligned reads (absolute numbers and percent of trimmed reads)	Unique alignments (absolute numbers and percent of aligned reads)	Non-duplicated aligned reads (absolute numbers and percent of aligned reads)	Reads aligning to ribosomal RNA (absolute numbers and percent of trimmed reads)
BN-03 liver	37,870,875 (96.4%)	32,882,723 (86.8%)	14,091,152 (37.2%)	805,959 (2.1%)
BN-03 heart left ventricle	34,925,794 (96.6%)	30,737,415 (88.0%)	13,866,921 (39.7%)	211,474 (0.6%)
BN-04 liver	39,082,130 (96.8%)	34,683,358 (88.7%)	15,931,304 (40.8%)	181,485 (0.5%)
BN-04 heart left ventricle	38,793,389 (96.6%)	33,970,899 (87.6%)	14,413,293 (37.2%)	53,931 (0.1%)
SHR-02 liver	31,681,912 (95.4%)	27,079,893 (85.5%)	10,814,241 (34.1%)	151,962 (0.5%)
SHR-02 heart left ventricle	32,713,041 (95.0%)	28,471,299 (87.0%)	12,435,350 (38.0%)	284,145 (0.8%)
SHR-03 liver	29,610,437 (95.5%)	25,567,357 (86.3%)	10,308,328 (34.8%)	201,844 (0.7%)
SHR-03 heart left ventricle	30,803,403 (94.94%)	27,026,756 (87.7%)	12,470,719 (40.4%)	65,253 (0.2%)
Illumina human body map 2.0 liver (SRA accession number ERR030895)	45,896,887 (94.5%)	42,741,402 (93.1%)	10,370,858 (22.6%)	548,720 (1.1%)

To quantify the de novo detection of splice sites, we applied the FindCirc pipeline [5], which identifies back-splicing events indicative of circular RNAs. Comparing the long RNA reads presented here with the smaller RNA fragments that were sequenced alongside the ribosome protected fragments [2], we found the following improvements in the average numbers of detected back-splice junctions per sample: for liver samples, 273 vs. 38, and for heart left ventricle, 679 vs. 61.

In summary, we provide here a high quality RNA sequencing dataset for two rat strains, with two different tissues from two different animals for each strain. This data can be used to analyze differences between tissues and strains, e.g. variant calling, de novo transcriptome assembly, differential expression and splicing, or circRNA detection. Since the samples originate from the same animals as the ones in the study about translational differences between a healthy rat (BN-*Lx*) and a model for hypertensive cardiomyopathy and metabolic syndrome (SHR) [2], the ribosome profiling data from that study can readily be integrated into the analysis.

## Limitations

- The data described here is limited to two organs, heart left ventricle and liver.
- The sequencing depth does not allow for reliable quantification and characterization of very low abundant transcripts.
- Genomic DNA was not sequenced, and has to be used from other animals of the same strains.

## Abbreviations

BN-*Lx*: brown Norway rat strain carrying the polydactyly-luxate syndrome *Lx* allele
SHR: spontaneous hypertension rat
SRA: short read archive
